# 3DSNP 2.0: update and expansion of the noncoding genomic variant annotation database

**DOI:** 10.1093/nar/gkab1008

**Published:** 2021-11-01

**Authors:** Cheng Quan, Jie Ping, Hao Lu, Gangqiao Zhou, Yiming Lu

**Affiliations:** Beijing Institute of Radiation Medicine, State Key Laboratory of Proteomics, Beijing 100850, China; Beijing Institute of Radiation Medicine, State Key Laboratory of Proteomics, Beijing 100850, China; Beijing Institute of Radiation Medicine, State Key Laboratory of Proteomics, Beijing 100850, China; Beijing Institute of Radiation Medicine, State Key Laboratory of Proteomics, Beijing 100850, China; Beijing Institute of Radiation Medicine, State Key Laboratory of Proteomics, Beijing 100850, China

## Abstract

The rapid development of single-molecule long-read sequencing (LRS) and single-cell assay for transposase accessible chromatin sequencing (scATAC-seq) technologies presents both challenges and opportunities for the annotation of noncoding variants. Here, we updated 3DSNP, a comprehensive database for human noncoding variant annotation, to expand its applications to structural variation (SV) and to implement variant annotation down to single-cell resolution. The updates of 3DSNP include (i) annotation of 108 317 SVs from a full spectrum of functions, especially their potential effects on three-dimensional chromatin structures, (ii) evaluation of the accessible chromatin peaks flanking the variants across 126 cell types/subtypes in 15 human fetal tissues and 54 cell types/subtypes in 25 human adult tissues by integrating scATAC-seq data and (iii) expansion of Hi-C data to 49 human cell types. In summary, this version is a significant and comprehensive improvement over the previous version. The 3DSNP v2.0 database is freely available at https://omic.tech/3dsnpv2/.

## INTRODUCTION

With the aid of high-throughput chromosome conformation capture (3C) technologies, such as Hi-C (1) and ChIA-PET (2), we previously developed a database named 3DSNP for annotating the regulatory function of human noncoding variants by exploring their three-dimensional (3D) interactions with genes mediated by chromatin loops (3). Since its publication in 2017, a number of paradigm-shifting biotechnologies, such as single-molecule long-read sequencing (LRS) (4,5) and single-cell assay for transposase accessible chromatin sequencing (scATAC-seq) (6), have been widely applied in genetic studies, providing critical information that was previously missing from the annotation of human noncoding variants.

Characterization of the full spectrum of genetic variation is critical to understanding human health and disease. The structural variation (SV) is an important type of genetic variant, typically encompassing inversions, deletions, duplications, and insertions of ≥50 base pairs (bp) in length. Most SVs are located in noncoding genomic regions and have been shown to affect more of the genome per nucleotide change than any other class of sequence variant (7). In addition, SVs are often involved in large deletion, insertion and duplication regions on chromosomes (8,9), leading to significant alterations of local chromatin architecture (10).

The identification of SVs with short-read sequencing (SRS) has been very challenging, especially for SVs located in repetitive genomic regions. Long-read sequencing, such as PacBio single-molecule real-time (SMRT) sequencing and nanopore sequencing, has been widely used to resolve SVs (4). Compared to SRS, LRS enables the resolution of SVs with much higher accuracy and sensitivity. For example, the Human Genome Structural Variation Consortium (HGSVC) generated fully phased genome assemblies for 35 individuals (32 unrelated and three children from parentṇchild trios) and identified 107 590 SVs, of which 68% were not discovered with SRS (11). However, as an increasing number of SVs have been identified by LRS studies, a systemic functional annotation platform for SVs is still lacking.

Identification of the functional target cell types/subtypes of a genetic variant is critical for the further exploration of its downstream biological functions. However, this is a very challenging task because of the high heterogeneity of the tissue microenvironment (12). Distal DNA regulatory elements such as enhancers and insulators exhibit significantly higher cell-type specificity (13), further promoting the need for a high-resolution annotation platform across tissues and cell types. The rapid development of single-cell omics, such as scATAC-seq, offers us an opportunity to address this issue. scATAC-seq traces the profiles of chromatin accessibility surrounding the variants in single cells (14), thus shedding light on the biological effects of genomic variants at single-cell resolution. Several large-scale scATAC-seq studies have been applied to map genome-wide chromatin accessibility across a variety of fetal and adult human tissues (15,16), providing valuable resources for the annotation of genetic variants.

As a result, we systemically updated our 3DSNP server to accommodate these advances. First, we systemically annotated SVs from a full spectrum of functions. In particular, taking advantage of the Hi-C datasets collected in our server, we assessed the effects of SVs on 3D chromatin architectures across a variety of cell types using well-established methods (17–19). Second, we displayed the accessible chromatin peaks surrounding the variants at single-cell resolution so that the target cell types/subtypes of a genetic variant could be systemically assessed. Third, we updated all the major contents of 3DSNP, including Hi-C, dbSNP and expression quantitative trait loci (eQTL), considering that the available Hi-C or ChIA-PET datasets have increased dramatically and that other databases integrated by 3DSNP, such as dbSNP (20) and GTEx (21), have also been greatly updated since 2017. In summary, the 3DSNP v2.0 version is a more useful database for noncoding variant annotation in the new era of long-read sequencing and single-cell omics.

## NEW FEATURES AND UPDATED CONTENT

### Functional annotation of structural variation

To annotate SVs, we first obtained a total of 107 590 SVs from the Human Genome Structural Variation Consortium (HGSVC v2) (11). All these SVs were robustly identified using fully phased genome assemblies. Similar to the SNP annotation strategy, SVs were systemically annotated from a variety of aspects, including 3D interacting genes, expression quantitative trait loci (eQTLs), chromatin state, transcription factor binding sites (TFBS), sequence motif alterations and evolutionary conservation. Notably, the SV-eQTLs were identified by HGSVC using the transcriptomes of 427 donors, which is different from the SNP-eQTLs obtained from GTEx. For each SV, we also calculated the linkage disequilibrium (LD)-associated SNPs and SVs using the HGSVC SV genotypes called from the 1000G project samples. To generate a functional overview, each SV was scored based on its annotated records for six functional categories: 3D interacting genes, enhancer state, promoter states, transcription factor binding sites, altered sequence motifs and conservation scores.

**Figure 1.**
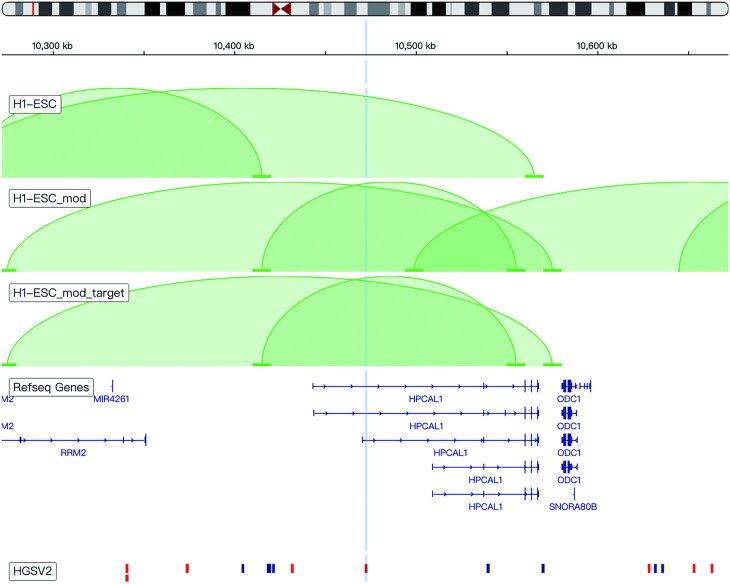
Effects of structural variations (SVs) on local chromatin architecture. From top to bottom, the three chromatin loop tracks represent (i) the normal chromatin loops without considering any SV in the genome, (ii) the altered chromatin loops considering all possible SVs located in the relevant topologically associating domain (TAD) at the population scale and (iii) the altered chromatin loops considering only the presence of the query SV. The two tracks below the chromatin loop tracks are the RefSeq gene track and HGSVC v2 structural variation track. Red blocks in the SV track indicate deletions; blue blocks in the SV track indicate insertions; and the vertical blue line indicates the query SV.

Notably, the new platform enables the measurement of the potential effects of SVs on local chromatin architecture. Using a published algorithm focusing on the prediction of chromatin architecture alterations by SVs (17), we calculated three different sets of genome-wide chromatin loops for each Hi-C map across the 49 human cell types: (i) the normal chromatin loops without considering any SV in the genome, (ii) the altered chromatin loops considering all possible SVs located in the relevant topologically associating domain (TAD) at the population scale and (iii) the altered chromatin loops considering only the presence of the query SV to block any interferences from adjacent SVs, as shown in Figure 1.

In addition, we extracted reliable SVs detected by short-read sequencing from ClinVar and added them to the final SV set for systemic annotation. In fact, there were in total 92 505 SVs in the ClinVar collection, including duplications, insertions, deletions, and inversions. Among these SVs, 38 584 were not collected by dbSNP, and only 1259 had a length longer than 50 bp. That is, most novel SVs are short indels beyond the scope of our study. After filtering by the review status, which should be ‘criteria_provided’, ‘multiple_submitters’ or ‘reviewed_by_expert_panel’, only 727 SVs were added to our database, 44 of which were already in the HGSVC. After integration, we annotated these new SVs and recalculated the scores for all SVs in 3DSNP v2.

### Annotation of SNP or SV target cell types/subtypes using scATAC-seq data

**Figure 2.**
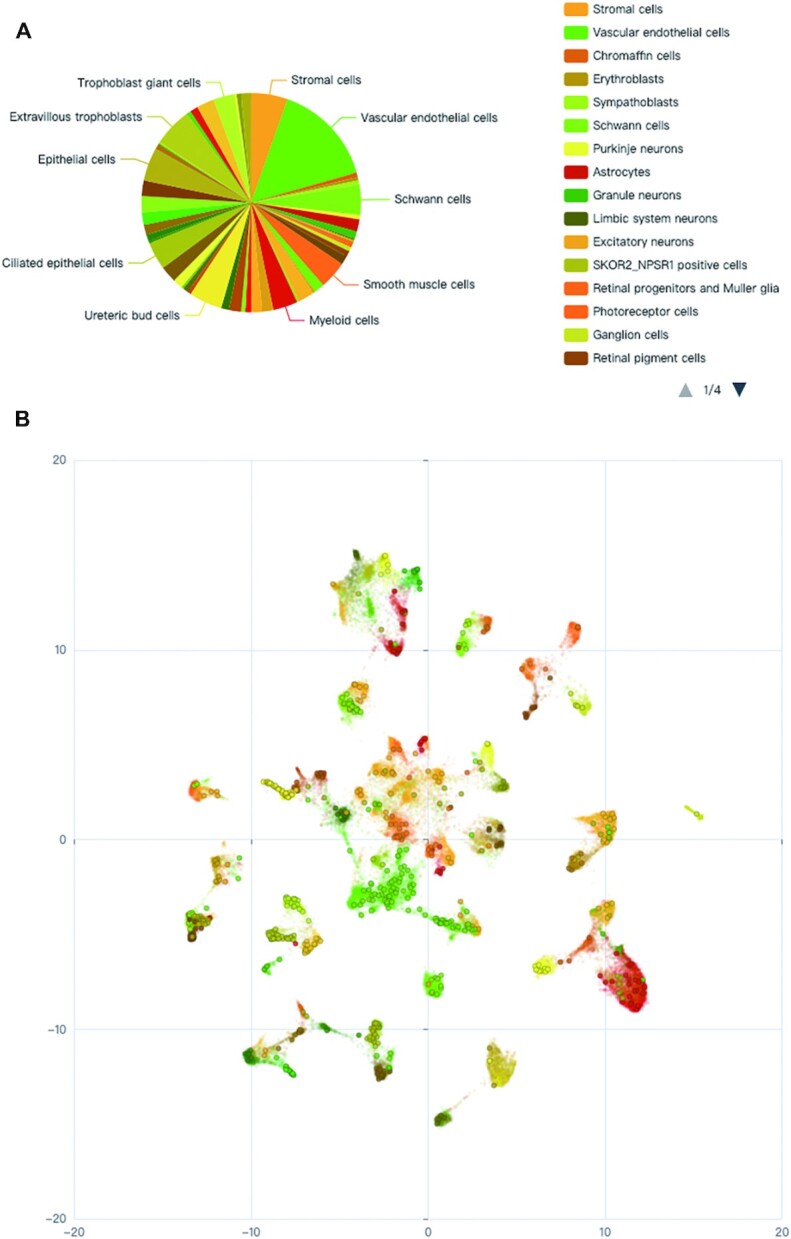
Annotation of SNP or SV target cell types/subtypes using scATAC-seq data. (**A**) Pie chart of the percentages of cells with the nearest accessible chromatin peaks across 126 cell types/subtypes in 15 fetal tissues. (**B**) The Uniform Manifold Approximation and Projection (UMAP) plot of an open chromatin region across single cells. A total of 86 685 single cells across 126 cell types/subtypes are plotted. Single cells are represented by scattered hollow circles; cells with open chromatin states of the peak are marked with solid circles; and different colors represent the 126 cell types/subtypes.

A measurement of local chromatin accessibility at the single-cell level will greatly facilitate the prioritization of functional target cell types/subtypes of a genetic variant. As a result, we collected two publicly available scATAC-seq datasets, including 53 scATAC-seq samples from 15 human fetal tissues (16) and 70 samples from 25 adult tissues (15). For the fetal scATAC-seq dataset, a total of 1 001 437 ATAC peaks were identified genome-wide in 86 685 single cells, which were classified into 126 cell types/subtypes. The nearest ATAC peak (if any) within a 100 kb window surrounding a query SNP or SV was derived and its accessible states across all the cells were plotted using the Uniform Manifold Approximation and Projection (UMAP) (Figure 2). One can easily prioritize the potential target cell types/subtypes of the queried SNP or SV by looking up cell clusters with high rates of open chromatin states across their single cells. For the adult scATAC-seq dataset, a total of 756 414 open chromatin regions termed *cis*-regulatory elements (cCREs) were obtained in 472 373 single cells, which were classified into 54 cell types/subtypes. Since the raw *peaks* × *cells* matrix is not yet available, we used the average cCRE score of each peak across cells within the same cell type/subtype. For each SNP or SV query, the potentially functional cell types were sorted based on the accessibility scores of the cCREs overlapping the variant.

### Population genetic statistics

With our better understanding of multivariant adaptation, recent studies discovered that SNPs and SVs could jointly participate in the evolution of the genome and contribute to environmental adaptation. To avoid recombination with maladapted genomic backgrounds, candidate adaptive variants discovered thus far often occur in noncoding regions. Combining SNP genotypes from 1000G and SV genotypes from HGSVC, we searched for positive natural selection signals among the five major populations (AMR, Admixed American; EAS, East Asian; EUR, European; AFR, African and SAS, South Asian). Population genetic statistics were calculated between each population and the others, including fixation index statistics (*F*_ST_), integrated haplotype homozygosity scores (iHS) and cross-population number of segregating sites by length (XPNSL).

### Content updates

*Additional cell types/tissues with Hi-C data*. The number of available cell types with Hi-C data increased from 12 to 49, so substantially more 3D interacting genes can be identified for a query variant in the updated version.

*Latest dbSNP version*. The dbSNP database (20) was updated from version 146 to 154, with the number of annotated SNPs increasing from 149 254 102 to 700 385 017.

*Latest GTEx database*. The GTEx database (21) was updated from version 6 to 8, with the number of significant SNP-gene pairs increasing from 19 582 729 to 71 478 528.

## INTERFACE

### New interactive interface for visualizing chromatin architecture and accessibility

**Figure 3.**
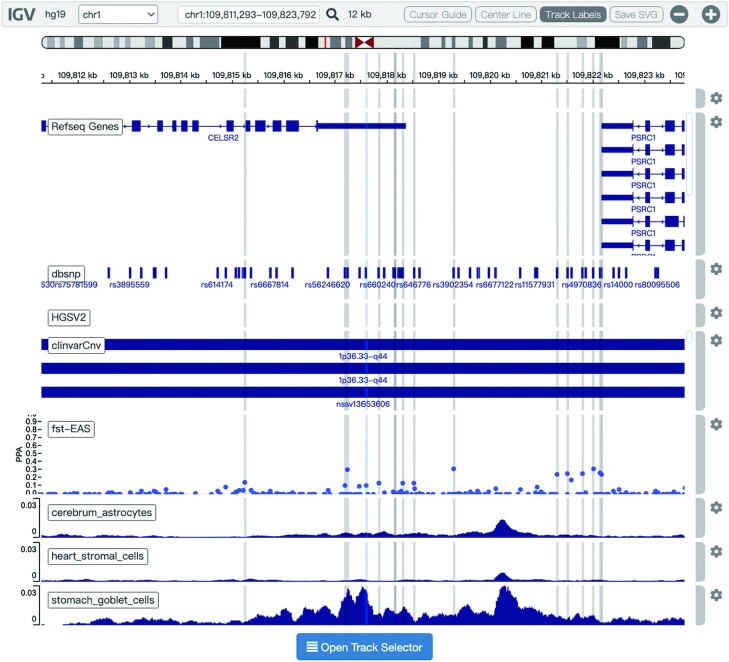
New interface for visualizing chromatin architecture and accessibility surrounding the query variant. The IGV.js plugin is utilized to interactively display multiple genomic features in different tracks. The query SNP is marked by a vertical blue line; LD-linked SNPs of the query are marked by vertical gray lines. From top to bottom, the tracks represent: adjacent RefSeq genes, adjacent SNPs, structural variation in HGSVC2 or ClinVar CNV, fixation index statistics *F*_ST_ of surrounding 1000G SNPs, and scATAC-seq peaks averaged across cells for three cell types in three fetal tissues.

The former version of 3DSNP provided a series of user-friendly interfaces for users to search, browse, visualize, and export the results. To facilitate the visualization of the local chromatin architecture alteration and chromatin accessibility surrounding query SNPs or SVs, we used the IGV.js plugin (22,23) to replace the former USCS genome browser screenshots, as shown in Figure 3. Six major annotation categories were integrated in this platform: basic annotation, Hi-C loops, scATAC-seq, variant types, variant pathogenicity, and population genetics. Available tracks in the ‘basic annotation’ category include RefSeq genes, DNase sites and repetitive genomic regions identified by RepeatMasker (24); tracks in the ‘Hi-C loops’ category include the normal Hi-C loops, the altered loops by all SVs and the altered loops only by the query SV in 49 human cell types; tracks in the ‘scATAC-seq’ category include averaged chromatin accessibility profiles of 126 cell types/subtypes in 15 human fetal tissues; tracks in the ‘variant types’ category include SNVs and SVs collected from the dbSNP and HGSVC2 databases, respectively; tracks in the ‘variant pathogenicity’ category include ClinVar SNV, ClinVar CNV, ClinGen Haploinsufficiency, ClinGen Triplosensitivity and ClinGen Validity; and tracks in the ‘population genetics’ category include *F*_ST_, iHS and XPNSL. Users can conveniently select tracks of interest by clicking the ‘Track Selector’ button at the bottom of the tracks.

## DISCUSSION

Long-read sequencing technologies such as PacBio SMRT and nanopore enable the characterization of genetic variation in a much broader spectrum. With the aid of these technologies, SV has recently emerged as a hotspot of genetic research. Since most SVs are located in noncoding genomic regions, the interpretation of their functions is largely dependent on their possible alteration of epigenetic markers, chromatin accessibility and 3D chromatin architectures. Taking advantage of the annotation framework of noncoding SNPs, 3DSNP v2.0 can provide a full spectrum of functional annotations of SVs, as well as their potential effects on chromatin architectures.

In the future, other long-read technologies such as optical mapping (Bionano) will contribute substantially to the deposition of SVs. To incorporate these data, first, it is important to keep the database framework open and flexible for all types of variants. Expansion from SNV to SV annotation has demonstrated the good flexibility of the 3DSNP framework in incorporating new data. Second, as the cost of long-read technologies continues to drop, population-scale studies will enable a more robust identification of SVs and their genetically associated variants. Third, SV pathogenicity and the associations of SVs with human diseases should be systemically integrated to aid the identification and interpretation of clinically relevant SVs.

In addition, single-cell omics enables the characterization of genetic variation at much higher resolution. For example, scATAC-seq could characterize the genome-wide profiles of chromatin accessibility in single cells, providing a unique opportunity for the identification of the functional target cell types/subtypes for a SNP or SV. In the future, larger-scale scATAC-seq studies will resolve finer maps of the cellular microenvironment of heathy and disease-associated tissue samples, leading to more accurate identification of the target cell types/subtypes of genetic variants.

## DATA AVAILABILITY

3DSNP v2.0 is freely available at https://omic.tech/3dsnpv2/. The API for 3DSNP 2.0 is currently available at https://www.omic.tech/wordpress/3dsnp-v2-0-api/ and users can also download the entire database at https://www.omic.tech/wordpress/3dsnp-v2-0-data-download/.
